# Exploring the causal relationship between Helicobacter pylori infection and cervical spondylosis based on the Mendelian randomization method

**DOI:** 10.1097/MD.0000000000047485

**Published:** 2026-01-30

**Authors:** Changsui Yu, Xiaofeng Zhang, Zhongbao Yu, Shuren Wang, Kejian Lu, Fengyuan Zhan, Xinyue Zhang, Daoxiong Gong, Liguo Zhu, Junchen Li

**Affiliations:** a The Second Department of Spine, Wangjing Hospital of China Academy of Chinese Medical Sciences, Beijing, China; b Department of Traditional Chinese Osteopathy and Traumatology, Beijing Electric Power Hospital, Beijing, China; c The Second Affiliated Hospital of Heilongjiang University of Chinese Medicine, Harbin, China; d Chinese Medicine Department, Liaoning Yu Zhongbao Clinic of Traditional Chinese Medicine, Kuandian, China; e Department of Orthopedics, First Affiliated Hospital, Heilongjiang University of Chinese Medicine, Harbin, China; f Faculty of Information Technology, Beijing University of Technology, Beijing, China.

**Keywords:** causality, cervical spondylosis, Helicobacter pylori infection, Mendelian randomization

## Abstract

Currently, an association between Helicobacter pylori (Hp) infection and cervical spondylosis has been found in clinical practice. However, it is unclear whether there is a causal relationship between the two. To reveal the causal relationship between Hp infection and cervical spondylosis, we performed a two-sample two-way Mendelian randomization (MR) analysis. This study was based on a two-sample Mendelian randomized design with a genome-wide association study of Hp infection and cervical spondylosis, systematically screened for genetic instrumental variables (IV), applied inverse-variance weighted (IVW), MR-Egger regression and weighted median estimator (WME), simple mode (SM), weighted mode (WM) to assess the pathogenic association between the 2 diseases, and sensitivity analyses were used to further validate the robustness of the results. The results of forward MR analysis with Hp infection as the exposure were (OR [95% CI] = 1.110 [0.970–1.271], *P* = .166), and the results of reverse MR analysis with cervical spondylosis as the exposure were (OR [95% CI] = 0.226 [0.023–2.161], *P* = .253). The results showed no significant association between Hp infection and cervical spondylosis at the gene level (*P* >.05). Sensitivity analyses were consistent with the results of the main analysis, confirming the robustness of the study. This study confirms that there is no causal relationship genetically between Hp infection and cervical spondylosis.

## 1. Introduction

As a common spinal disorder, cervical spondylosis has a significant impact on the quality of life of patients, and its pathogenesis involves multiple factors that are not yet fully understood.^[1]^ In recent years, the association between inflammatory responses triggered by chronic infections and various diseases has attracted much attention, and Helicobacter pylori (Hp) infection, as a globally widespread chronic infection, may be associated with a variety of digestive and cardiovascular diseases.^[2]^

Hp infection induces a systemic inflammatory response in the body, and the release of inflammatory factors may affect vascular endothelial function, alter the rheological properties of the blood, and promote abnormalities in bone metabolism.^[3]^ Given that the development of cervical spondylosis is closely related to pathological processes such as cervical disc degeneration, vertebral osteophytes, and peripheral vascular-neurological compression, and that inflammation may play an important role,^[4]^ it is hypothesized that there may be some potential causal association between Hp infection and cervical spondylosis.

However, traditional observational studies are susceptible to confounding factors and reverse causality, making it difficult to conclusively confirm the causal relationship. The Mendelian randomization (MR) method, which uses genetic variation as an instrumental variable (IV), can effectively overcome these limitations and provides new opportunities and avenues for in-depth investigation of the causal link between Hp infection and cervical spondylosis.^[5]^ The aim of this study was to analyze whether there is a causal relationship between Hp infection and cervical spondylosis by using MR, with a view to opening up new perspectives on the etiology of cervical spondylosis and providing a scientific basis for its prevention and treatment strategies.

## 2. Information and methodology

### 2.1. Research design

In this study, we explored the causal relationship between genetically predicted Hp infection and cervical spondylosis by a bidirectional two-sample MR method. Genetic variants strongly associated with Hp infection were screened as IVs to explore their causal impact on cervical spondylosis. In addition, reverse MR analyses assessed the possibility of reverse causality. MR analyses followed 3 core assumptions^[6]^: the correlation hypothesis: genetic IV is strongly associated with exposure factors; the independence hypothesis: genetic IV is independent of confounding factors affecting “exposure-outcome”; and the exclusivity hypothesis: genetic IV can only affect outcome through exposure.

### 2.2. Data sources

Pooled data related to Hp infection was obtained from Butler-Laporte et al^[7]^ published in 2020, it included 9,170,312 single nucleotide polymorphisms (SNPs), 8735 sample size. Data related to cervical spondylosis included in this study was obtained from Zhenxiao Ren et al^[8]^ published in 2024, it included 16,380,237 SNPs, case group 171,956, control group 328,044. There were 16,380,237 SNPs in this dataset, 17,1956 in the case group and 328,044 in the control group. The above data are from the European population, and the sexes are male and female.

### 2.3. Instrumental variables

After obtaining data from the website using R software, to avoid analytical bias due to strong linkage disequilibrium between SNPs, the screening criteria were as follows^[9]^: *P* <5 × 10^−8^; physical distance *M* >10,000 kb between every 2 genes; and *r*^2^ threshold of linkage disequilibrium between genes <0.001, *F* value >10, and ultimately obtain SNPs that are independent of each other and significantly correlated with exposure factors, which were used as final IV.

### 2.4. MR analysis

The statistical software R 4.3.1 was used in this study with a test level of α = 0.05. The main methods were inverse-variance weighted (IVW), MR-Egger regression, weighted median estimator (WME), simple mode (SM) and weighted mode (WM). The IVW method, as the most dominant analytical method for MR, was developed by calculating the Wald ratios corresponding to individual SNPs and weighting and combining the Wald ratios of all SNPs. The MR-Egger regression method provides relatively robust estimates independent of IV validity and can be used to detect the presence of horizontal pleiotropy using an intercept test. The WME method produces a more robust estimate of the causal effect of ineffective IVs. Two other MR methods were added to the scatterplot analysis in this study, including the SM and WM methods. The WM method requires a smaller sample size than the other methods, while ensuring a smaller error and lower error rate. The SM method is able to group SNPs with similar causal effects together and make causal inferences based on the similarity of the estimated effects.^[10]^

### 2.5. Sensitivity analysis

In this study, Cochran *Q* test based on the IVW method was used to explore the presence of heterogeneity, with *P* <.05 as the criterion for determining the level of heterogeneity, in addition, MR-Egger regression analysis was used to assess the possibility of horizontal pleiotropy, which was indicated when *P* <.05. Finally, a leave-one-out sensitivity analysis was also performed to assess the robustness of the results when individual SNPs were removed.^[11]^

## 3. Results

### 3.1. SNP information for instrumental variables

In this study, Hp infection and cervical spondylosis were used as exposure factors, respectively, and SNP loci of genome-wide significance were screened according to the screening criteria using the R software, and 10 and 7 SNPs were obtained as IV, respectively (see Table S1, Supplemental Digital Content, https://links.lww.com/MD/R291), which illustrates comprehensive information on SNPs associated with Hp infection; see Table S2, Supplemental Digital Content, https://links.lww.com/MD/R291), which illustrates comprehensive information on SNPs associated with cervical spondylosis).

### 3.2. Forward MR analysis

#### 3.2.1. *MR analysis results*

**Figure 1.**
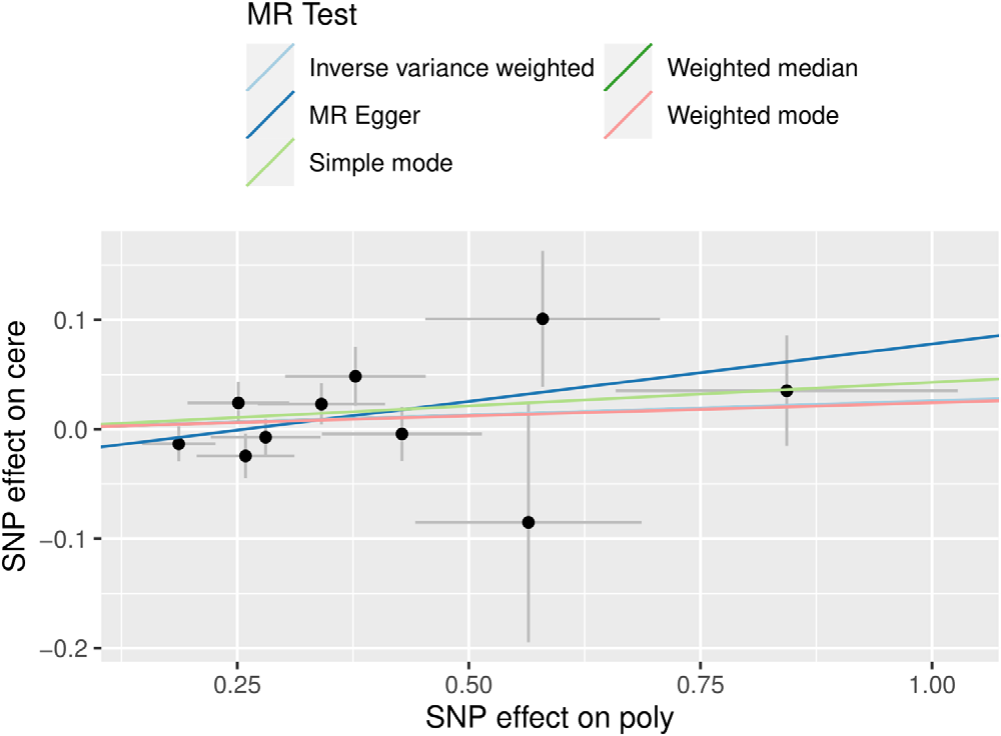
Scatterplot of forward MR (forward Mendelian randomized scatterplot of Helicobacter pylori infection and cervical spondylosis). MR = Mendelian randomization.

MR analysis was performed using MR-Egger, WME, IVW, SM, and WM analyses from the TwoSampleMR package, which showed ORs and 95% CIs of 1.110 (0.970–1.271), 1.025 (0.964–1.091), 1.026 (0.977–1.077), 1.043 (0.951–1.145), and 1.024 (0.939–1.117). From the results, it can be seen that the *P*-values of the 5 tests of MR-Egger, WME, IVW, SM, and WM were .166, .414, .296, .386, and .595, respectively, and the results were >.05, and the differences were not statistically significant (Table 1), so the perspective of MR analysis can be concluded that there is no causal relationship. The results were visualized (Fig. 1).

**Table 1:** Forward MR results of Hp infection on cervical spondylosis.

Method	nsnp	β	SE	*P*-*val*	OR (95% CI)
MR Egger	10	0.105	0.069	.166	1.110 (0.970–1.271)
WME	10	0.025	0.031	.414	1.025 (0.964–1.091)
IVW	10	0.026	0.024	.296	1.026 (0.977–1.077)
SM	10	0.042	0.047	.386	1.043 (0.951–1.145)
WM	10	0.024	0.044	.595	1.024 (0.939–1.117)

CI = confidence interval, Hp = Helicobacter pylori, IVW = inverse-variance weighted, MR = Mendelian randomization, OR = odds ratio, SM = simple mode, WM = weighted mode, WME = weighted median estimator.

#### 3.2.2. *Sensitivity analysis results*

**Figure 2.**
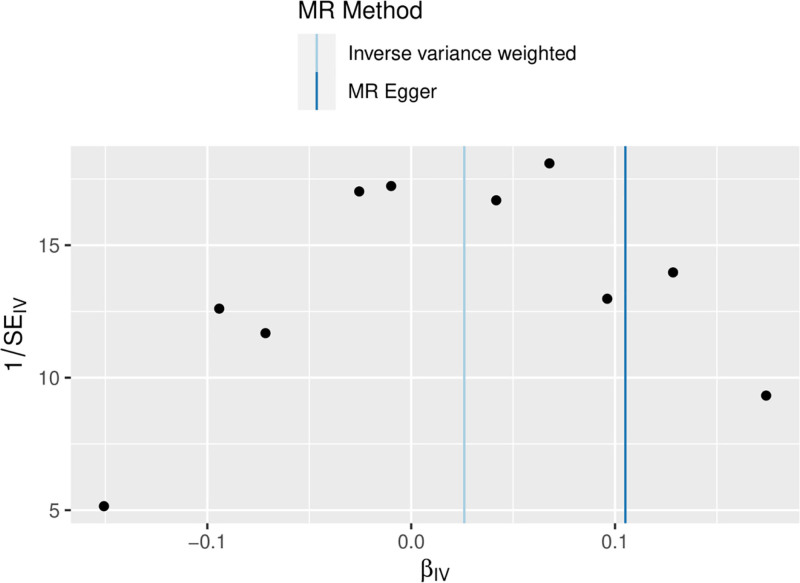
Funnel plot of the results of the heterogeneity test for forward MR method analysis. MR = Mendelian randomization.

In this study, the screening criteria for IV were strictly followed and the population of the same species was included, so the possibility of false-negative results was unlikely. The results were tested for heterogeneity, and the *Q*-value and QP-value of MR-Egger and IVW were 9.269 (.320) and 11.001 (.275), respectively, which were >.05 (Table 2), indicating that there was no Heterogeneity. The results were visualized (Fig. 2).

**Table 2:** Results of the forward MR heterogeneity test.

Method	*Q*	*Q*_df	*Q_*P-val
MR Egger	9.269	8	.320
IVW	11.001	9	.275

IVW = inverse-variance weighted, MR = Mendelian randomization.

The intercept of MR-Egger regression was used to verify the presence of polyvalence in the study, and its results showed that the value of Egger-intercept was −0.027, close to 0, SE = 0.022, *P* = .256, which means that there is no horizontal polyvalence, and that there is no interference of the results of MR with multiple effects.

**Figure 3.**
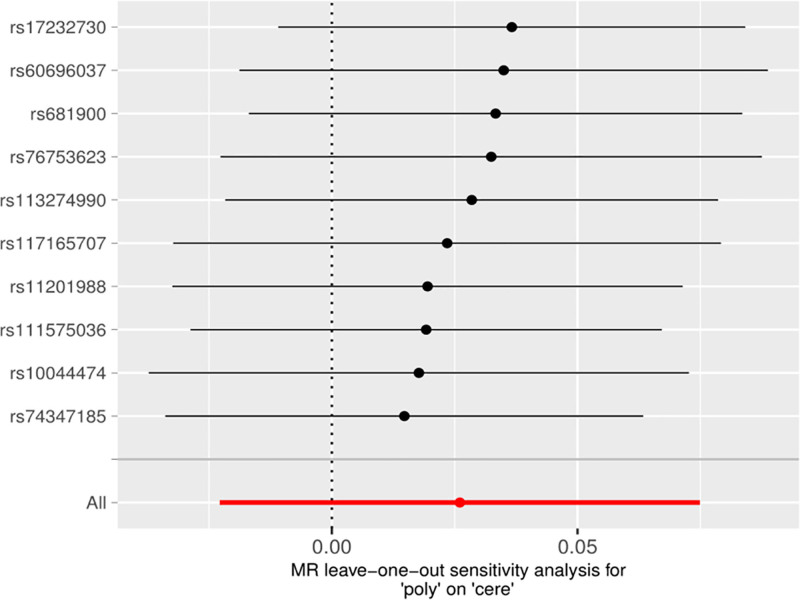
Forward MR “Leave-one-out” sensitivity analysis results. MR = Mendelian randomization.

The sensitivity analysis “Leave-one-out” method was used to visualize the results of the IVW method, and after removing single SNPs sequentially, the IVW effect values of the remaining SNPs did not show large fluctuations, and they were all close to the red dots in the graphs, which indicated that there were no SNPs in the IV that had a strong effect on the results, and showed that the results obtained by the previous method were stable and trustworthy. The results obtained by the IVW method are stable and reliable (Fig. 3).

### 3.3. Reverse MR analysis

**Figure 4.**
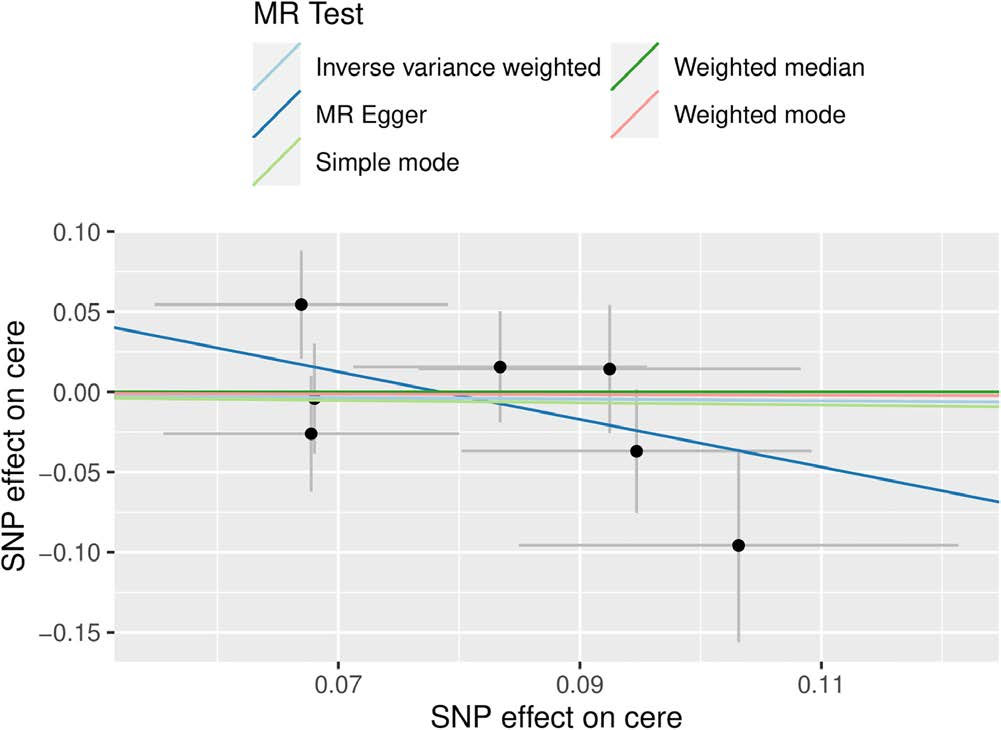
Scatterplot of reverse MR (reverse Mendelian randomized scatterplot of Helicobacter pylori infection and cervical spondylosis). MR = Mendelian randomization.

**Figure 5.**
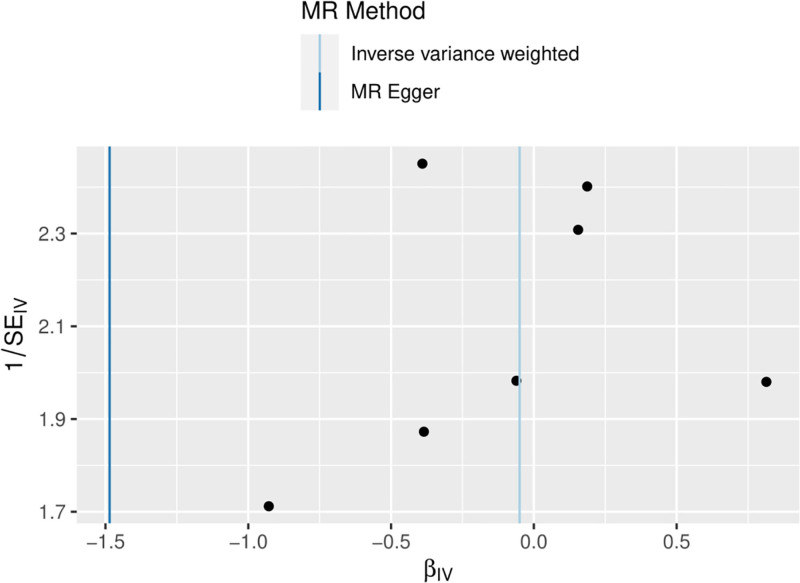
Funnel plot of the results of the heterogeneity test for reverse MR method analysis. MR = Mendelian randomization.

**Figure 6.**
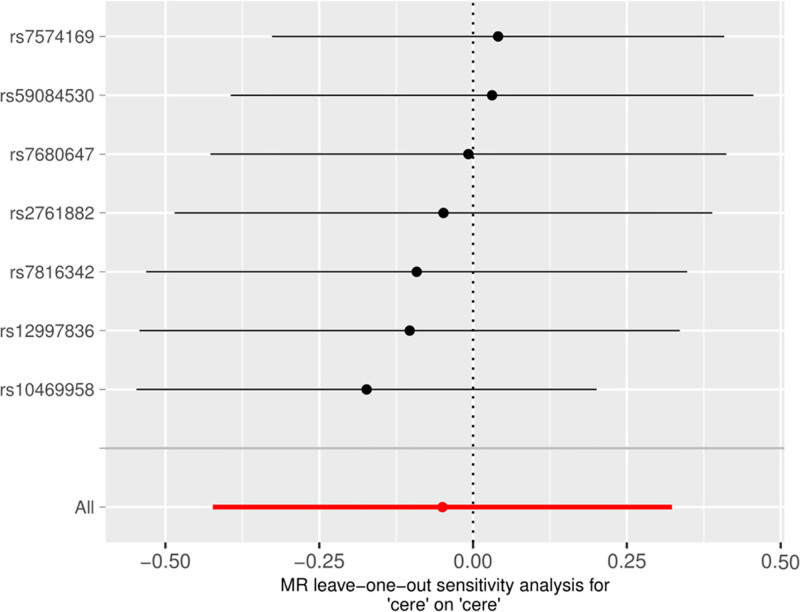
Reverse MR “Leave-one-out” sensitivity analysis results. MR = Mendelian randomization.

In the reverse MR analysis, cervical spondylosis was used as an exposure and Hp infection as an outcome, and the same SNP screening conditions were set as in the forward MR, and 7 SNPs were finally included as IVs in the reverse MR analysis. The results of the MR analysis showed no causal relationship between cervical spondylosis and Hp infection (MR-Egger, OR [95% CI] = 0.226 [0.023–2.161], *P* = .253; WME, OR [95% CI] = 1.000 [0.619–1.613], *P* = .999; IVW, OR [95% CI] = 0.951 [0.655–1.381], *P* = .793; SM, OR [95% CI] = 0.928 [0.459–1.877], *P* = .843; WM, OR [95% CI] = 0.980 [0.538–1.785], *P* = .951) (Table 3 and Fig. 4). The results were tested for heterogeneity, and the *Q*-values and *QP*-values of IVW and MR–Egger were 6.825 (.337) and 5.175 (.394). The results of the pleiotropy test showed SE = 0. 092, *P* = .262. Sensitivity analysis did not detect any heterogeneity as well as the presence of horizontal pleiotropy (Fig. 5), and the leave-one-out method did not reveal any aberrant SNPs (Fig. 6).

**Table 3:** Reverse MR results of cervical spondylosis on Hp infection.

Method	nsnp	β	SE	*P*-val	OR (95% CI)
MR Egger	7	−1.485	1.151	.253	0.226 (0.023–2.161)
WME	7	9.25 × 10^−05^	0.244	.999	1.000 (0.619–1.613)
IVW	7	−0.049	0.190	.793	0.951 (0.655–1.381)
SM	7	−0.074	0.359	.843	0.928 (0.459–1.877)
WM	7	−0.019	0.305	.951	0.980 (0.538–1.785)

CI = confidence interval, Hp = Helicobacter pylori, IVW = inverse-variance weighted, MR = Mendelian randomization, OR = odds ratio, SM = simple mode, WM = weighted mode, WME = weighted median estimator.

## 4. Discussion

Hp infection of the human body triggers a series of complex pathophysiological processes.^[12]^ Long-term Hp infection often leads to a state of chronic inflammation with a sustained release of inflammatory factors into the blood circulation.^[13]^ These inflammatory factors can reach all parts of the body, including the cervical spine region, with the blood circulation.^[14]^ In the cervical region, inflammatory factors may interfere with the normal metabolism of cervical disc cells and accelerate disc degeneration.^[15]^ Intervertebral disc degeneration leads to a decrease in its height and elasticity, which in turn affects the stability of the cervical spine.^[16]^ In order to maintain the stability of the cervical spine, the muscles, ligaments and other soft tissues around the vertebrae will experience compensatory tension and spasm, and long-term muscle tension and spasm will lead to muscle fatigue and injury, further aggravating the biomechanical imbalance of the cervical spine.^[17]^ At the same time, inflammatory factors may also stimulate cervical vertebral osteophytes, and the proliferated bone may compress the surrounding structures such as nerves and blood vessels, triggering a series of cervical spondylosis symptoms, such as neck pain, numbness of the upper limbs, and dizziness.^[18]^ In addition, the systemic inflammatory response triggered by Hp infection may also affect the local microcirculation of the cervical spine, reducing the supply of nutrients and the removal of metabolic wastes, resulting in a decrease in the repair capacity of the cervical spine tissues, making them more susceptible to lesions and injuries, and thus facilitating the development of cervical spondylosis.^[19]^ However, this study did not find a direct causal association between Hp infection and cervical spondylosis, which may be due to the small sample size of this study, which resulted in insufficient statistical efficacy and limited variation explained by genetic variations, and the fact that the sample of this study mainly originated from the European region, and the results of this study may be affected by the unique dietary, behavioral and other lifestyles of the local population. Moreover, many previous positive studies of Hp infection and cervical spondylosis were based on observational epidemiological and case-control studies.^[20]^ From the perspective of biological mechanisms, the systemic inflammatory response triggered by Hp infection may be a key factor leading to the development of cervical spondylosis.^[21]^ It has been demonstrated that Hp infection can induce the body to produce a variety of inflammatory mediators, which may reach the cervical spine through the blood circulation, thereby affecting the microenvironment of cervical spine tissues, promoting disc degeneration, vertebral osteophytes, and inflammatory changes in the surrounding soft tissues, and ultimately increasing the risk of cervical spondylosis.^[22]^ In summary, although there is no causal relationship between Hp infection and cervical spondylosis, it can still aggravate the condition of cervical spondylosis and indirectly increase the incidence of cervical spondylosis, and the early detection of Hp infection may help to prevent cervical spondylosis from occurring and indirectly reduce the incidence of cervical spondylosis.^[23]^

The MR design of this study has significant advantages over previous traditional observational studies. Traditional observational studies are often confounded by confounding factors and reverse causality, making it difficult to conclusively determine causality.^[24]^ For example, although some previous cohort-based or case-control studies have suggested a possible association between Hp infection and cervical spondylosis, it is not possible to rule out the influence of confounding factors, such as lifestyle and dietary habits, on the outcomes.^[25]^ The present study, however, utilizes genetic variation as an IV, which largely avoids these problems and makes causal inferences more reliable. However, there may be some differences in effect size estimation between our findings and some traditional studies, which may be due to differences in research methodology and differences in genetic background and environmental factors of different study populations. Despite the innovative nature of this study, there are still some limitations. Firstly, MR analysis relies on specific genetic assumptions, such as the validity of IV and the absence of pleiotropy. Although we have screened and validated the IV as much as possible during the study, we still cannot completely exclude the influence of potential genetic pleiotropy on the results.^[26]^ Secondly, this study is only based on the existing genetic database and statistical analysis methods, which may have omitted important genetic information or statistical errors. In addition, the complex biological pathway between Hp infection and cervical spondylosis was only preliminarily explored in this study, and the specific molecular mechanisms and signaling pathways have not yet been analyzed in depth.

## 5. Conclusion

The results of this study are potentially instructive for clinical practice. If the causal relationship between Hp infection and cervical spondylosis is further confirmed, appropriate attention should be given to the screening, diagnosis, and treatment of Hp infection, in addition to focusing on localized cervical spine lesions and traditional risk factors, in the preventive and therapeutic strategies for cervical spondylosis. Particularly in regions or populations with a high prevalence of Hp infection and a high incidence of cervical spondylosis, early intervention of Hp infection may help to reduce the risk of developing cervical spondylosis or delay its progression. Future studies could further expand the sample size to include more ethnically and geographically diverse populations to increase the generalizability of the findings. At the same time, in-depth basic experimental research will be conducted to elucidate the detailed molecular mechanism of cervical spondylosis caused by Hp infection, and to explore specific therapeutic targets and interventions for Hp infection-associated cervical spondylosis, so as to provide new ideas and methods to improve the prognosis of patients with cervical spondylosis.

## Author contributions

**Conceptualization:** Changsui Yu.

**Data curation:** Changsui Yu, Shuren Wang, Kejian Lu, Liguo Zhu, Junchen Li.

**Formal analysis:** Daoxiong Gong.

**Funding acquisition:** Changsui Yu, Xiaofeng Zhang.

**Methodology:** Zhongbao Yu.

**Software:** Xinyue Zhang.

**Supervision:** Fengyuan Zhan.

**Writing – original draft:** Junchen Li.

## Supplementary Material


